# Converging evidence for a two-domain framework of microvascular dysfunction in sepsis

**DOI:** 10.1186/s13054-026-06253-w

**Published:** 2026-08-12

**Authors:** Alexandros Rovas, Philipp Kümpers

**Affiliations:** Department of Medicine D, Division of General Internal and Emergency Medicine, Nephrology, and Rheumatology, University Hospital Münster, Albert-Schweitzer-Campus 1, Münster, 48149 Germany

**To the Editor**,

Beltrán et al. recently advanced pediatric sepsis phenotyping by integrating clinical variables, circulating biomarkers, and quantitative sublingual videomicroscopy within an unsupervised machine-learning framework [1]. Unlike previous pediatric classifications based predominantly on systemic inflammation, clinical trajectories, or organ dysfunction, their analysis incorporated the microcirculation as a defining biological component. It identified three phenotypes—Preserved, Partially Compensated, and Decompensated—with distinct clinical, inflammatory, and microvascular profiles, as well as stepwise increases in persistent organ dysfunction and mortality [1].

Importantly, these phenotypes differed in both overall disease severity and the balance between the two complementary domains of microvascular dysfunction: endothelial surface layer (including the endothelial glycocalyx) and capillary density. These two domains were captured by GlycoCheck-derived sublingual measurements, with the perfused boundary region (PBR) providing a functional readout of endothelial surface layer, and capillary density reflecting the availability of the perfused capillary exchange network. Across the Preserved, Partially Compensated and Decompensated phenotypes, progressive impairment of both domains occurred alongside increasing clinical severity [1]. Therefore, this study is novel not only in identifying distinct pediatric phenotypes with different prognoses, but also in demonstrating that microvascular heterogeneity is embedded within the biological organization of sepsis.

We believe that these findings gain additional significance when interpreted alongside the recently published EDGE study by Scarbeck et al., which examined adults presenting to the emergency department with suspected infection [2]. At first glance, the two studies appear fundamentally different. They enrolled different patient populations, captured different stages of disease, applied different analytical strategies, and examined different clinical endpoints. Beltrán et al. identified latent phenotypes using unsupervised machine learning and related them to persistent organ dysfunction and mortality in critically ill children [1]. In contrast, the EDGE study evaluated a prospective adult emergency department cohort and used baseline PBR and capillary density to define microvascular profiles that were subsequently related to disease progression and adverse outcomes [2].

Despite these differences, both studies assessed the sublingual microcirculation using the same videomicroscopy platform and, when interpreted together, point to a remarkably similar biological observation: the most adverse clinical trajectories were characterized by concurrent endothelial surface layer impairment and capillary density reduction.

What is remarkable is not that two independent studies identified prognostically relevant microvascular phenotypes, but that they converged on a shared framework for organizing septic microvascular dysfunction. This convergence raises an important question: Do endothelial surface layer impairment and capillary density reduction reflect different manifestations of the same pathological process, or distinct biological domains of septic microvascular dysfunction? We favour the latter interpretation.

Accordingly, we suggest that the findings of both studies are best interpreted within a two-domain endothelial–microvascular framework comprising two partially distinct yet interacting domains of vascular dysfunction. By biological domains, we refer to complementary aspects of microvascular biology that are governed by partly distinct mechanisms and can be interrogated by different imaging-derived parameters. One domain reflects endothelial surface layer properties, functionally approximated by alterations in PBR. The second reflects the functional availability of the perfused capillary exchange network, represented by capillary density. Importantly, these domains should not be regarded as interchangeable measurements of the same pathological process. Rather, they reflect different aspects of endothelial–microvascular biology that may deteriorate simultaneously, sequentially, or independently within individual patients (Fig. 1).

**Fig. 1:**
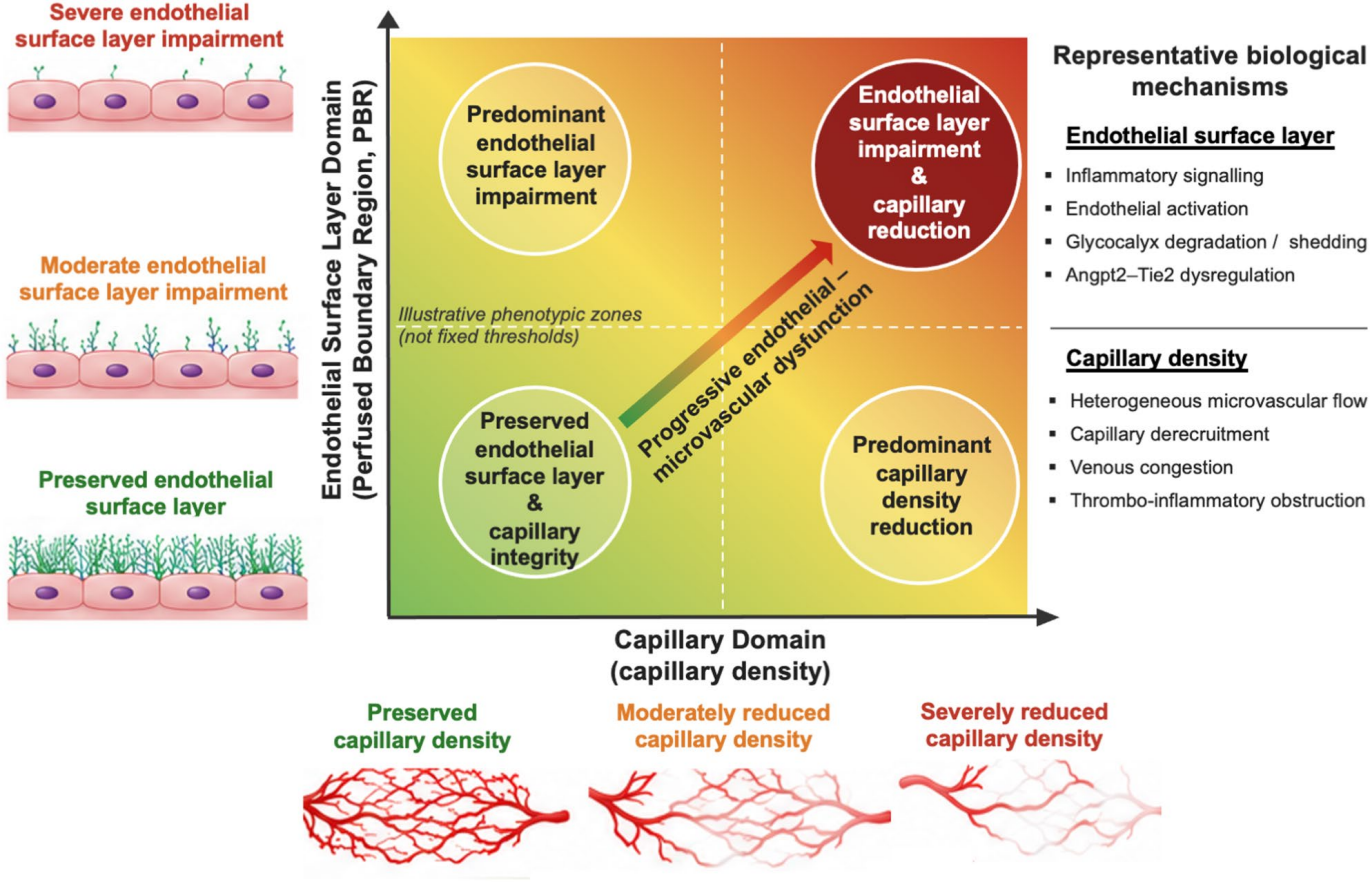
Two-domain framework of septic microvascular dysfunction assessed by sublingual GlycoCheck videomicroscopy. The framework integrates two complementary microvascular domains: endothelial surface layer impairment, functionally reflected by increasing perfused boundary region (PBR), and capillary density reduction, reflected by decreasing density of the perfused capillary network. The background gradient illustrates increasing endothelial–microvascular dysfunction from preserved integrity to combined impairment. Dashed lines schematically indicate illustrative boundaries separating phenotypic zones. Colored circles denote representative phenotypes: intact microvasculature (lower left), isolated impairment of either domain (upper left/lower right), and combined endothelial–microvascular dysfunction (upper right). The diagonal arrow depicts progressive deterioration. Insets illustrate representative alterations of endothelial surface layer and capillary density

Viewed from this perspective, the apparent differences between the two studies become complementary. Beltrán et al. adopted a data-driven approach, enabling clinically relevant microvascular phenotypes to emerge from multidimensional clustering. In contrast, the EDGE study conducted an exploratory analysis that stratified patients according to baseline endothelial surface layer and capillary density characteristics. Despite their fundamentally different analytical strategies, both studies independently described a comparable biological continuum of endothelial–microvascular dysfunction. In both studies, preserved endothelial–microvascular integrity was associated with the most favorable clinical trajectories, whereas concurrent endothelial surface layer impairment and capillary density reduction characterized the most adverse states. Taken together, these independent observations support a shared conceptual framework in which septic microvascular dysfunction is best understood as the interaction of two complementary biological domains rather than a single homogeneous process.

The biological plausibility of this interpretation is supported by the partly distinct regulatory mechanisms governing endothelial surface properties and capillary density. Increased PBR may occur in the context of endothelial activation, inflammatory signaling, enzymatic glycocalyx degradation, and dysregulation of endothelial-stabilizing pathways such as angiopoietin–Tie2 [3–6]. Importantly, PBR should not be interpreted as a direct measure of glycocalyx thickness, endothelial permeability, or plasma leakage, but rather as a functional readout of changes affecting the endothelial surface layer. Conversely, capillary density reflects the availability of the perfused capillary exchange network and is influenced by heterogeneous blood flow, impaired vasomotor regulation, capillary derecruitment, venous congestion, thrombo-inflammatory obstruction, leukocyte–platelet interactions, and microvascular flow heterogeneity [7–13]. Although these mechanisms overlap biologically, they are not obligatorily coupled, providing a plausible explanation for why endothelial surface layer impairment and capillary density reduction may coexist, but not necessarily evolve in parallel.

Importantly, our intention is not to redefine the phenotypes described by Beltrán et al., but to offer a biological interpretation of why they emerge. Clinical phenotypes describe observable patterns of disease expression, whereas biological domains may explain the regulatory mechanisms underlying these patterns. From this perspective, endothelial surface layer properties and capillary density should not simply be viewed as additional prognostic biomarkers, but as imaging-derived readouts of two interacting biological domains whose combined dysfunction is associated with adverse clinical trajectories across different patient populations, disease stages, and analytical approaches.

Whether these biological domains also represent distinct therapeutic targets remains unknown. Nevertheless, interpreting septic microvascular dysfunction through this framework may help integrate observations across different studies while providing a biologically plausible basis for future mechanistic investigations, biomarker development, and interventional trials. Viewed together, the studies by Beltrán et al. and Scarbeck et al. provide converging evidence that septic microvascular dysfunction is best understood not as a single homogenous process, but as the interplay between two partially distinct endothelial–microvascular domains.

## Data Availability

No datasets were generated or analysed during the current study.
